# Association of *PON1* gene promoter DNA methylation with the risk of Clopidogrel resistance in patients with coronary artery disease

**DOI:** 10.1002/jcla.22867

**Published:** 2019-03-19

**Authors:** Jia Su, Jiyi Li, Qinglin Yu, Xiaofeng Xu, Jingqiao Wang, Jin Yang, Xiaojing Li, Xiaomin Chen

**Affiliations:** ^1^ Department of Cardiology Ningbo No. 1 Hospital Ningbo Zhejiang China; ^2^ Department of Traditional Chinese Internal Medicine Ningbo No. 1 Hospital Ningbo Zhejiang China; ^3^ Department of Cardiology Ningbo Hospital of Zhejiang University Ningbo Zhejiang China

**Keywords:** clopidogrel resistance, coronary artery disease, DNA methylation, *PON1*

## Abstract

**Background and Aims:**

The failure of therapeutic response to clopidogrel in platelet inhibition, which is called clopidogrel resistance (CR), is more likely to cause cardiovascular events. We aimed to study the contribution of promoter DNA methylation of paraoxonase 1 (*PON1*) to the risk of clopidogrel poor response.

**Methods:**

Through VerifyNow P2Y12 assay, patient’ platelet functions were measured. Among 57 non‐CR and 49 CR patients, the levels of DNA methylation in four CpG dinucleotides on the *PON1* promoter were tested using bisulfite pyrosequencing technology. Besides, the relative expression of *PON1* mRNA was analyzed by quantitative real‐time PCR. Logistic regression was applied to investigate the interaction of *PON1* methylation and clinical factors in CR.

**Results:**

In the subgroup with dyslipidemia, we discovered that higher CpG4 levels of the *PON1* promoter indicated a poorer clopidogrel response (cases versus controls (%): 51.500 ± 14.742 vs 43.308 ± 10.891, *P* = 0.036), and the *PON1* mRNA expression was reduced in CR patients. Additionally, the logistic regression indicated that higher level of albumin and the index of ALT were related to a lower risk of CR, and the index of AST as well as the quantity of stent may be positively associated with CR.

**Conclusions:**

The DNA methylation of CpG4 in the *PON1* promoter would lead to a low expression of *PON1* mRNA, which might induce clopidogrel resistance in the patients with dyslipidemia, and the number of stents might be a risk for CR.

## 1 INTRODUCTION

Atherosclerotic plaque rupture or erosion, platelet activation and its aggregation, as well as thrombosis, are the main pathophysiological events involved in the progression from stable coronary artery disease (SCAD) to acute coronary syndrome (ACS).^1^ Dual antiplatelet treatment (usually using aspirin along with *P2Y12* receptor inhibitors, such as clopidogrel) has been the cornerstone treatment in patients after percutaneous coronary intervention (PCI). The above drugs, such as clopidogrel, could inhibit the ADP receptor, preventing sustained platelet aggregation, and thus lower cardiovascular risk.^2^

The response to clopidogrel varies greatly in different patients who undergo PCI,^3^ and various patients continue to afford adverse cardiovascular risk (10%‐40%).^4^ This clinical phenomenon has been correlated with the failure of therapeutic response to clopidogrel in platelet inhibition, which is called clopidogrel poor response or clopidogrel resistance (CR).^5^ In China, although the application of ticagrelor (a newer and stronger P2Y12 receptor inhibitor) reveals more consistent and rapid antiplatelet effect among ACS patients,^6^ the united major and minor PLATO bleeding risk was rising by 11%.^7^ Recently, the PHILO study found that event rates of primary safety and efficacy endpoints were higher in ticagrelor‐treated patients compared with clopidogrel‐treated ACS patients from Japan, Taiwan, and South Korea.^8^ The Korea Acute Myocardial Infarction Registry‐National Institute of Health (KAMIR‐NIH) study also reported that, compared with treatment using aspirin with clopidogrel, aspirin with prasugrel or aspirin with ticagrelor revealed close all‐cause mortality rates but higher bleeding risk.^9^ Hence, clopidogrel might be better than ticagrelor in treatment of East Asian ACS patients.

In continuing to prescribe clopidogrel for antiplatelet treatment, we should be more aware of the potential for CR; however, the pathological mechanism of CR remains unclear. Genetic or nongenetic factors may result in the different platelet activities, consisting of drug‐drug interactions,^10^ diabetes mellitus (DM),^11^ and so on. Moreover, intrinsic factors, particularly the expression of the *PON1* gene, were probably to affect clopidogrel's response. Bouman et al^12^ investigated *PON1* QQ192 homozygous individuals and found that they suffered a considerably higher risk for stent thrombosis, lower PON1 plasma activity, lower plasma concentrations of active metabolites, and lower platelet inhibition than RR192 homozygous patients. But, the result was contradicted by a meta‐analysis.^13^ Moreover, since various studies focused on single nucleotide polymorphisms, some had shifted their attention to epigenetics, such as DNA methylation, lncRNA, and cirRNA. Among them, DNA methylation is a stable and reliable epigenetic marker, which is occurred in the region of cytosine‐phosphate‐guanine (CpG) dinucleotide.^14^ Due to hypermethylation in CpG islands (CGIs), the gene expression is more likely to be transcriptional silencing,^15^ so as to regulate the activity of proteins. Currently, the effect of *PON1* gene DNA methylation on the clopidogrel resistance is poorly understood. Hence, in this study, we attempted to investigate whether DNA methylation of selected CpG islands in the *PON1* promoter is involved in clopidogrel resistance in Chinese CAD patients treated with clopidogrel.

## 2 METHOD

### 2.1 Study population

From 2012 to 2017, 106 acute coronary syndrome (ACS) patients were recruited at Ningbo NO. 1 Hospital. These patients were Han Chinese in eastern coast city of China. The inclusion criteria were as follows: (a) according to the recent ACC/AHA guidelines, ACS patients who underwent PCI using drug‐eluting stents, with most having multivessel disease of the coronary arteries or left main vessel disease; (b) patients who were administered 300 mg aspirin and 300 mg clopidogrel as a loading dose before PCI and received 100 mg aspirin and 75 mg clopidogrel daily as a maintenance dose; (c) patient older than 18 years, and (4) without aspirin resistance (ARU < 550). The exclusion criteria were as follows: (a) rheumatological disorders; (b) abnormal hepatic or kidney function; (c) active bleeding history; (d) concomitant treatment by warfarin or glycoprotein IIb/IIIa inhibitors; (e) recent or chronic clopidogrel treatment; and (f) the platelet was less than 150 000 μL or more than 500 000 μL.

The study protocol was reviewed and approved by the Ethics Committee at Ningbo NO. 1 Hospital and conformed to the principles outlined in the Declaration of Helsinki. All patients or their guardians provided written informed consent.

### 2.2 Clinical data collection and platelet function measurements

Venous blood samples were collected in plain tubes, and biochemical markers, such as the values of TC, TG, LDL, HDL, GLU, HbA1c, ALT, AST, and BUN, were measured. All the detection applied the standard process provided by the manufacturers, and then, the raw data were stored into the databank.

The patients’ platelet function was tested at 30 days after PCI, when the platelet reactivity was much stable compared with those just post‐PCI.^16^ Using the double‐syringe technique, blood samples were collected. And to avoid spontaneous platelet activation, the first 2‐4 mL of free‐flowing blood was discarded. The platelet function measurements were measured using the VerifyNow P2Y12 assay (Accumetrics Inc., San Diego, CA), which was to evaluate the responsiveness to P2Y12 antagonists.^17^ This assay reported P2Y12 reaction units (PRU) as the result, and the PRU greater than 240 considered as the existence of clopidogrel resistance.^18^ What was more, this assay could evaluate the responsiveness to aspirin and the reported aspirin reaction units (ARU) greater than 550 considered as the existence of aspirin resistance.

### 2.3 Genomic DNA extraction and DNA methylation assay

The QIAamp DNA Blood Mini Kit (Qiagen, Hilden, Germany) was used to extract human genomic DNA from the leukocytes of peripheral blood samples. The DNA concentrations, which must be greater than 500 ng/μL, were quantified using the NanoDrop 1000 (NanoDrop, Wilmington, DE). The levels of DNA methylation in 4 CpG dinucleotides, which were located on the *PON1* gene promoter (GRCh37.p13:94954884‐94952884) (Figure S1), were determined using bisulfite pyrosequencing technology. The process of bisulfite pyrosequencing included sodium bisulfite DNA conversion chemistry using the EpiTech Bisulfite Kit (Qiagen, Valencia, CA), polymerase chain reaction (PCR) amplification using the PyroMark PCR Kit, as well as the targeted fragment sequencing using PyroMark Gold Q24 Reagents.^19^ Through PyroMark Assay Design software, the PCR and pyrosequencing primers were designed, and each of them is listed in Table S1.

### 2.4 Assay of PON1 mRNA

The RNA from peripheral blood samples was extracted using the RNeasy Plus Universal Kit (Qiagen), and 1 μg of RNA was applied to synthesize cDNA by PrimeScript™ RT Reagent Kit with gDNA Eraser (Takara Bio, Kusatsu, Japan). Template cDNAs were diluted 1:4, and the *PON1 *relative expression was quantified through the ABI 7500 Quantitative Real‐time PCR (qRT‐PCR) System (Applied Biosystems, Foster City, CA) and normalized with housekeeping gene GAPDH. The primers of qRT‐PCR amplification were designed using the software of Primer Premier 5, and their sequences are listed in Table S2. After samples run in triplicate, we received the mean value. The relative quantitative method was implemented for the calculation of the level on *PON1* mRNA.

### 2.5 Statistical analysis

Statistic breakdown was operated using PASW Statistics 18.0 version software (IBM, Chicago, Illinois, USA). All data for enumeration data were depicted as medians with interquartile ranges (IQRs), and measurement data were expressed as means ± standard deviation. A collection of statistical analysis was implemented to study the association with *PON1* DNA methylation, mRNA expression, clinical features, and clopidogrel resistance. We applied Fisher’s exact test or Pearson’s chi‐square to analysis the relationship between enumeration data and CR. Meanwhile, for measurement variables, we used Wilcoxon rank‐sum test or *t *test for unpaired samples. Multiple linear regression was used to investigate the effect between metabolic variables and *PON1* DNA methylation. Logistic regression was implemented to test the interrelation of *PON1* methylation and confounding factors in CAD patients with CR. It was determined statistically significant when two sides *P*‐value was less than 0.05.

## 3 RESULTS

### 3.1 Patients’ characteristics

A sum of 106 CAD patients met above requirements, and they were recruited in our present study. Among them, 49 patients were considered as suffering clopidogrel poor reactivity. This study cohort was similar to that in our former research,^19^ and the clinical characteristics and demographic were also similar. Except for albumin, the clinical variables were well matched. That indicated CR patients were more likely to have lower albumin levels.^19^

### 3.2 The relationship between clopidogrel resistance and PON1 methylation levels

**Figure 1:**
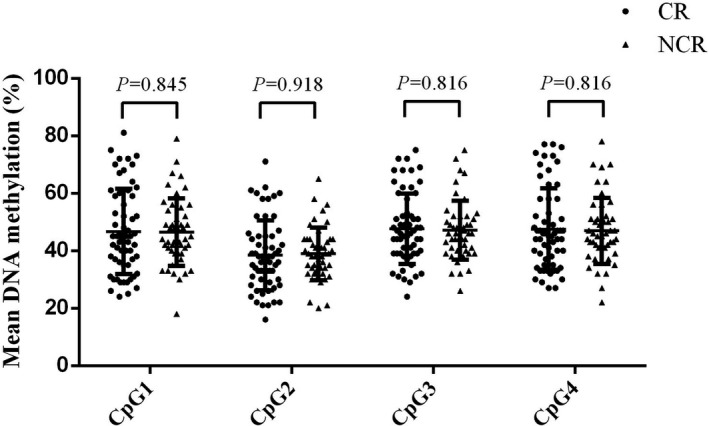
A comparison of PON1 gene promoter DNA methylation levels between cases and controls

In our study, we chosen a fragment (GRCh37.p13:94954884‐94952884) including four CpG dinucleotides. Via bisulfite pyrosequencing, we investigated the association of DNA methylation levels in *PON1* gene promoter among these CR and NCR patients. It is shown in Table 1 and Figure 1 that in selected fragments, the methylation levels of CpG1, CpG2, CpG3, and CpG4 in *PON1* were not significantly associated with CR.

**Table 1:** A comparison of PON1 gene promoter DNA methylation levels between cases and controls

	CR (49)	NCR (57)	*t*	*P*
CpG1	46.939 ± 14.429	46.421 ± 12.774	0.196	0.845
CpG2	38.551 ± 12.145	38.772 ± 9.822	‐0.104	0.918
CpG3	47.694 ± 12.326	47.175 ± 10.585	0.233	0.816
CpG4	47.980 ± 13.762	46.684 ± 12.407	0.510	0.611

CR, clopidogrel resistance; NCR, nonclopidogrel resistance.

**Figure 2:**
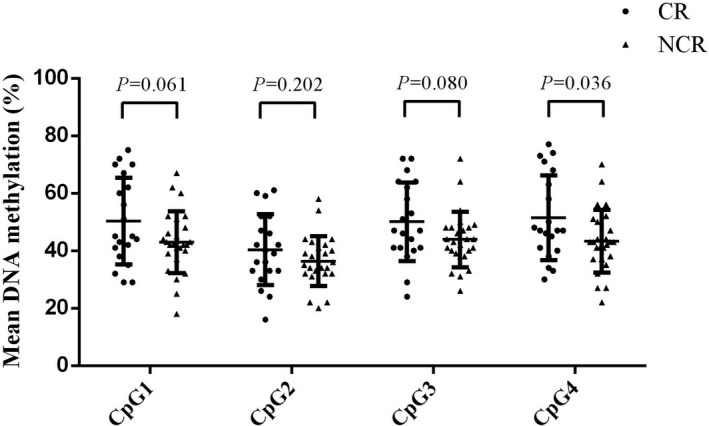
A comparison of PON1 gene promoter DNA methylation levels between cases and controls in the subgroup with dyslipidemia

Then, we conducted a subunit analysis by different clinical variables to assess whether the *PON1* gene promoter DNA methylation levels (containing CpG1, CpG2, CpG3, and CpG4) were associated with the clopidogrel resistance. We discovered that if patients had dyslipidemia, higher CpG4 indicated a poorer clopidogrel response (cases vs controls (%): 51.500 ± 14.742 vs 43.308 ± 10.891, *P* = 0.036), but there was no statistical significance in any other subgroups (Figure 2 and Table 2).

**Table 2:** A comparison of PON1 gene promoter DNA methylation levels between cases and controls in the subgroup with dyslipidemia

	CR (20)	NCR (26)	*t*	*P*
CpG1	50.300 ± 15.100	42.962 ± 10.776	1.924	0.061
CpG2	40.350 ± 12.330	36.346 ± 8.657	1.294	0.202
CpG3	50.100 ± 13.719	43.923 ± 9.625	1.795	0.080
CpG4	51.500 ± 14.742	43.308 ± 10.891	2.169	0.036

CR, clopidogrel resistance; NCR, nonclopidogrel resistance.

### 3.3 The relationship between clopidogrel resistance and *PON1 *mRNA expression

**Figure 3:**
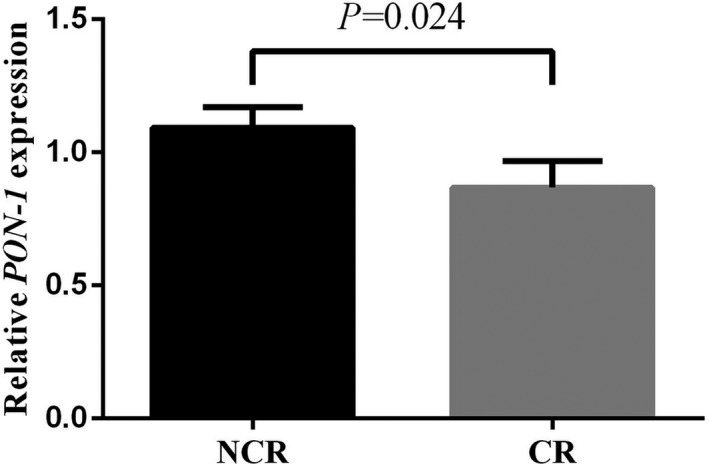
A comparison of PON1 mRNA expression between cases and controls in the subgroup with dyslipidemia

We tested the relative expression of PON1 mRNA through qRT‐PCR to determine whether a different PON1 expression could influence the various clopidogrel responses. Unexpectedly, the results were insignificant (Figure S2). However, in the subgroup with dyslipidemia, we discovered that PON1 mRNA expression was reduced in CR patients (Figure 3).

### 3.4 Multivariate regression analysis

Because DNA methylation might be influenced by our confounding factors, we implemented multiple linear regression to investigate the effect of clinical factors on DNA methylation; however, we observed that the association was not significant (*F* = 0.672, *F*‐value = 0.822, *R* ^2^ = 0.391).

Meanwhile, considering the effect of clinical variables on CR, we performed the logistic regression analysis with nongenetic and genetic variables. The results indicated that the indexes (such as ALT and albumin levels) were protective factors of clopidogrel resistance (Table S3). Additionally, the number of stents and the value of AST were correlated with CR (Table S3). Furthermore, since we carried out logistic regression analysis in dyslipidemia subgroup, the results showed that the quantity of stent was correlated to a poorer clopidogrel response (Table S4).

## 4 DISCUSSION

Antiplatelet therapy has been considered a research hotspot in the area of CAD treatment for a long time, whether on the topic of the choice of drug (clopidogrel or ticagrelor) or the time course of dual antiplatelet treatment. Although ticagrelor manifested more consistent, rapid, and effective platelet inhibition,^6^ there was a higher incidence of bleeding compared with clopidogrel.^7^ One recent COSTIC study published in ESC 2018 revealed that clopidogrel might be more suitable for the CAD patients in China. Therefore, the studies focused on the various responses to clopidogrel in Chinese patients are of vital significance.

The risk of CR was approximately 10%‐30%, and patients with CR were more likely to experience a thrombotic event.^3^ The phenomenon of clopidogrel resistance was influenced by many kinds of extrinsic factors (environment, comorbidities, drug interactions, and so on) and intrinsic factors that may contribute to CR.^20^ For instance, our former study indicated that the male gender, higher albumin in males, and hyperlipidaemia decreased the CR incidence.^21^ Another study found that chronic kidney disease (CKD) appeared to be related to a poor clopidogrel response and a higher risk of stent thrombosis in PCI patients with diabetes.^22^ In this study, after logistic regression analysis with extrinsic and intrinsic variables in the total population and the subgroup with dyslipidemia, we discovered that the quantity of stent was correlated with CR, which was similar to the findings in our former research.^19^ This finding might be due to coronary microvascular impairment in patients after (PCI), which would increase the platelet reactivity.^23^ Moreover, we found that liver function and albumin levels would affect the clopidogrel response. This might be due to the biological process related to the metabolism of clopidogrel in the liver, and thus, liver malfunction would affect the activity of the clopidogrel response. However, considering the limited sample size and unmeticulous stratification standards, these results should be taken cautiously. Furthermore, one study reported that there was insignificant association between smoking, dyslipidemia, diabetes, or utilization of nonsteroidal anti‐inflammatory drugs and CR in Saudi patients with coronary heart disease,^24^ which might be due to the different population and different environment. Hence, with larger sample sizes, additional researches might confirm the validity of our conclusions in later years.

Clopidogrel is a second‐generation *P2Y12* receptor inhibitor,^25^ and specific genetic variants are responsible for clopidogrel's transport (ATP‐binding cassette subfamily B member 1 [*ABCB1*]),^26^ metabolism (CYP enzymes^27^), and action (*P2Y12*,^19^ *PON‐1* 28). Among them, the paraoxonase 1 (*PON1*) gene might play a vital role in CR. The human paraoxonase 1 gene (*PON1*), located on the long arm of chromosome 7 at q21.3, has nine exons and eight introns.^29^ The *PON1 *gene, which is involved in the HDL antioxidative activity, forms part of a repertoire of HDL‐associated enzymes, such as platelet‐activating factor acetyl‐hydrolase and lecithin‐cholesterol acyltransferase.^30^ It has the ability to hydrolyze oxidized LDL cholesterol and cleave phospholipid peroxidation adducts, resulting in potential atheroprotective and cytoprotective effects.^31^ Several studies have indicated that due to increased oxidative stress and damage, reduced PON1 activity will influence serum glucose, increase the risk of diabetes mellitus,^32^ and lower platelet inhibition.^12^ Meanwhile, *PON1* participates in the process of clopidogrel esterification and its following inactivation,^33^ more likely leading to clopidogrel resistance.

A former study showed that single nucleotide polymorphisms of *PON1* gene were interrelated with lower clopidogrel responsiveness in atherosclerotic patients,^34^ and the *PON1 *Q192R polymorphism relies on potential association with clopidogrel biotransformation, which was an alternate pathway mediated by paraoxonase enzyme.^35^ However, the above conclusions have been challenged by a quantity of studies that failed to replicate these consequences,^36^ which might be due to epigenetic changes. Hence, we turned our focus to DNA methylation, and our present study found that in the group of CR patients with dyslipidemia, CpG4 of *PON1* hypermethylation and expression of *PON1* mRNA were lower. The outcome indicated that the levels of CpG4 DNA methylation in *PON1* promoter would lead to lower expression of *PON1* mRNA, which might induce the occurrence of clopidogrel resistance. It was the first research focused on the relationship between DNA methylation of *PON1 *promoter and clopidogrel resistance. A recent study investigated the role of intrinsic variables and the DNA methylation of CpG island in* PON1* promoter on clinical adverse events after dual antiplatelet treatment and found that hypomethylation of CpGs might be a weak risk for the event of bleeding^37^; however, they did not analyze the relationship with PRU, and they did not further test the mRNA expression. A larger sample size and a more advanced empirical approach would give us a chance to improve our limitations and further the exploration of the underlying mechanism.

Recently, evidence has indicated that the epigenetic modifications, including histone marks, DNA methylation, and long noncoding RNAs, were involved in various diseases, such as a lasting impairment of cardiovascular function.^38^ The DNA methylation that occurred within the range of cytosine‐phosphate‐guanine (CpG) dinucleotide, which did not lead to the DNA sequence changes, was a reliable epigenetic marker,^14^ and CGI hypermethylation was more likely to affect gene expression by transcriptional silencing and regulate protein synthesis.^39^ We discovered that some aberrant methylation was considered to participate in the occurrence and development of coronary artery disease,^40^ breast cancer,^41^ and psychotic disorders.^42^ For instance, *ABCA1* DNA methylation was a predictive biomarker for the CAD development and was independent of plasma lipid concentration.^40^ Since 2014, several studies have explored the influence of DNA methylation in *ABCB1*,^21^ *P2Y12*,^19^ and cytochrome *P450* enzymes^43^ on CR. This time, we chose *PON1* as the target gene and found some significant results. However, we need to be cautious concerning the results due to a limited sample size. Meanwhile, regardless of the above clopidogrel metabolic‐related genes, we should consider other genes and their epigenetic modifications that affect CR. Thus, our research’ conclusions ought to be taken with caution. In the future, we might try to apply DNA methylation chips to investigate the significant gene and enrichment analysis of possible pathways to further examine the mechanism of CR.

To our knowledge, the present study was the first research to examine the correlation of *PON1* promoter methylation and its mRNA expression with CR. Although considerable efforts were made during this research, there were some inherent limitations. First, we only selected one fragment of the CGI from the *PON1* gene promoter, and there probably be some other regions related to clopidogrel resistance. Second, functional experiments are needed to validate the molecular mechanisms of the *PON1* promoter methylation in CR, for example, validation on cell or animal levels. Third, unknown confounding factors, such as gene‐gene and gene‐environment coactions, might exist that change the gene expression and lead to biased results. However, exploring the molecular mechanisms of CR was worthwhile. Multicentre studies with large sample sizes are needed for further investigation and assessment.

In summary, this study indicates that the DNA methylation level of CpG4 in the *PON1* promoter would lead to a low expression of *PON1* mRNA and potentially induce the occurrence of clopidogrel resistance in patients with dyslipidemia. Additionally, the logistic regression analysis showed higher ALT and albumin values were correlated to a decreased incidence of CR, and the quantity of stent and the value of AST were positively correlated with CR. However, larger researches with a more advanced methods and more effective planning would further confirm the validity of our discovery and the evaluation of the pathogenesis of CR.
