# Supplementary material for: Association of PON1 gene promoter DNA methylation with the risk of Clopidogrel resistance in patients with coronary artery disease
Source: J Clin Lab Anal. 2019 Mar 19;33(5):e22867. doi: 10.1002/jcla.22867 (PMC6595294; doi:10.1002/jcla.22867)
Supplement: Supplementary file 1 [file JCLA-33-e22867-s001.doc]

Table S1 Primers for PON1 gene CpG island loci analysis

| Group | DNA sequence (5'→3') | Modification | Purification |
| --- | --- | --- | --- |
| Forward primer | GGGATTTTTGGTTGAAAGTGTTG |  | PAGE |
| Reverse primer | ACCACAACCCAACTTACTAATAAA | 5'Biotin | HPLC |
| Sequencing primer | TTGTAGGTAGGTAGAGT |  | PAGE |

Table S2 Primers of qRT-PCR amplification for *PON1* mRNA analysis

| Name | Group | Base sequence (5'→3') |
| --- | --- | --- |
| PON1 | Forward primer | CCACCAGTCTTCTTACCA |
|  | Reverse primer | AAGTCTTCAGAGCCAGTT |
| GADH | Forward primer | GGACCTGACCTGCCGTCTA |
|  | Reverse primer | AGGAGTGGGTGTCGCTGT |

PON1: paraoxonase 1; GADH: glyceraldehyde phosphate dehydrogenase.

Table S3 Logistic regression analysis with nongenetic and genetic factors in the total population

|  | B | Std. Error | Wald | ***P value*** | Exp (B) |
| --- | --- | --- | --- | --- | --- |
| CpG 1 | -0.009 | 0.096 | 0.009 | 0.923 | 0.991 |
| CpG 2 | 0.001 | 0.101 | 0.000 | 0.989 | 1.001 |
| CpG 3 | -0.216 | 0.13 | 2.736 | 0.098 | 0.806 |
| CpG 4 | 0.193 | 0.122 | 2.487 | 0.115 | 1.213 |
| Age | 0.023 | 0.039 | 0.349 | 0.555 | 1.023 |
| Gender(male) | 1.633 | 0.936 | 3.042 | 0.081 | 5.120 |
| Hypertension | -0.143 | 0.77 | 0.035 | 0.853 | 0.867 |
| Diabetes mellitus | -0.256 | 1.186 | 0.046 | 0.829 | 0.774 |
| Hyperlipidemia | -1.204 | 0.766 | 2.474 | 0.116 | 0.300 |
| Current smoking | -0.622 | 0.826 | 0.566 | 0.452 | 0.537 |
| Alcohol abuse | -1.384 | 1.047 | 1.747 | 0.186 | 0.251 |
| BMI | 0.007 | 0.136 | 0.003 | 0.957 | 1.007 |
| Sent | 1.570 | 0.549 | 8.184 | **0.004** | 4.806 |
| EF | 0.044 | 0.053 | 0.677 | 0.411 | 1.045 |
| TC | 0.516 | 0.576 | 0.805 | 0.370 | 1.676 |
| TG | -0.078 | 0.299 | 0.069 | 0.793 | 0.925 |
| HDL | 0.813 | 1.503 | 0.293 | 0.589 | 2.255 |
| LDL | -0.600 | 0.713 | 0.709 | 0.400 | 0.549 |
| Glu | 0.620 | 0.356 | 3.040 | 0.081 | 1.859 |
| HbA1c | -0.340 | 0.577 | 0.349 | 0.555 | 0.711 |
| ALT | -0.045 | 0.019 | 5.361 | **0.021** | 0.956 |
| AST | 0.007 | 0.003 | 4.976 | **0.026** | 1.007 |
| TBIL | 0.008 | 0.043 | 0.030 | 0.863 | 1.008 |
| A | -0.262 | 0.115 | 5.177 | **0.023** | 0.769 |
| BUN | -0.213 | 0.182 | 1.362 | 0.243 | 0.808 |
| CR | 0.014 | 0.02 | 0.483 | 0.487 | 1.014 |
| UA | -0.001 | 0.005 | 0.05 | 0.822 | 0.999 |
| hsCRP | 0.006 | 0.021 | 0.082 | 0.774 | 1.006 |
| PLT | 0.026 | 0.027 | 0.959 | 0.327 | 1.026 |
| MPV | 1.098 | 0.781 | 1.974 | 0.160 | 2.998 |
| PCT | -53.230 | 37.488 | 2.016 | 0.156 | 0.000 |
| PDW | 0.865 | 0.606 | 1.718 | 0.190 | 2.375 |
| Constant | -14.659 | 13.978 | 1.100 | 0.294 |  |

BMI: Body mass index; EF: Ejection fraction; TC: Total cholesterol; TG: Triglyceride; HDL: High density lipoprotein; LDL: Low densith lipoprotein; Glu: Glucose; HbA1c: Glycosylated haemoglobin A1c; ALT: Alanine aminotransferase; AST: Aspartic aminotransferase; TBIL: Total bile acids; A: Albumin; BUN: Blood urea nitrogen; CR: Creatinine: UA: Uric acid; hsCRP:; PLT: Platelet; MPV: Mean platelet volume; PCT: Plateletocrit; PDW: Platelet volume distribution width.

Table S4 Logistic regression analysis with nongenetic and genetic factors in the subgroup with dyslipidaemia

|  | B | Std. Error | Wald | P value | Exp (B) |
| --- | --- | --- | --- | --- | --- |
| CpG 4 | 0.060 | 0.031 | 3.674 | 0.055 | 1.061 |
| Sent | 1.007 | 0.462 | 4.763 | **0.029** | 2.738 |
| ALT | -0.032 | 0.024 | 1.833 | 0.176 | 0.968 |
| AST | 0.005 | 0.004 | 2.133 | 0.144 | 1.005 |
| A | -0.163 | 0.104 | 2.472 | 0.116 | 0.850 |
| Constant | 3.472 | 4.327 | 0.644 | 0.422 | 32.192 |

ALT: Alanine aminotransferase; AST: Aspartic aminotransferase; A: Albumin.


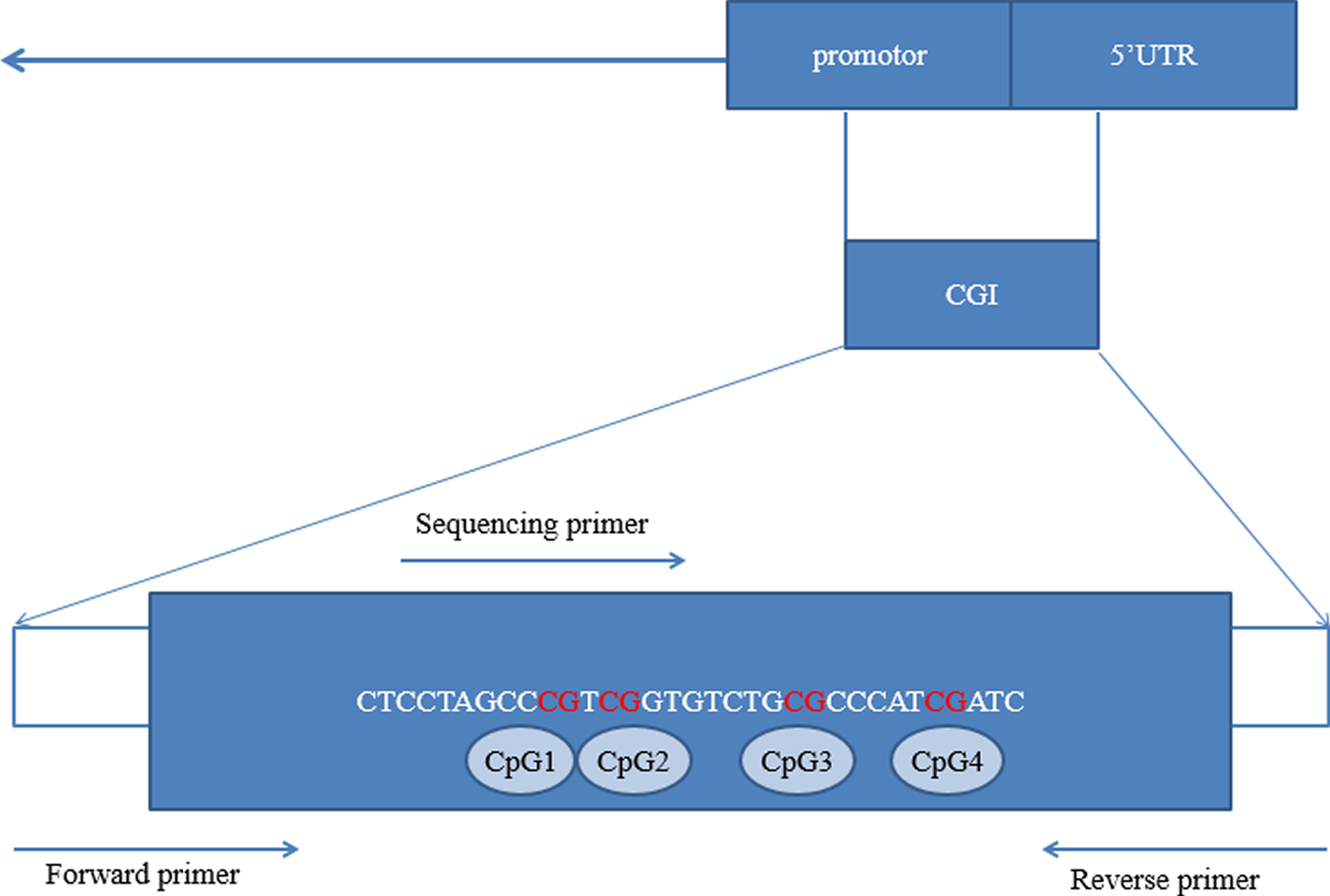


Figure S1Four CpGs in the PON1 gene promoter(GRCh37.p1394954884-94952884)


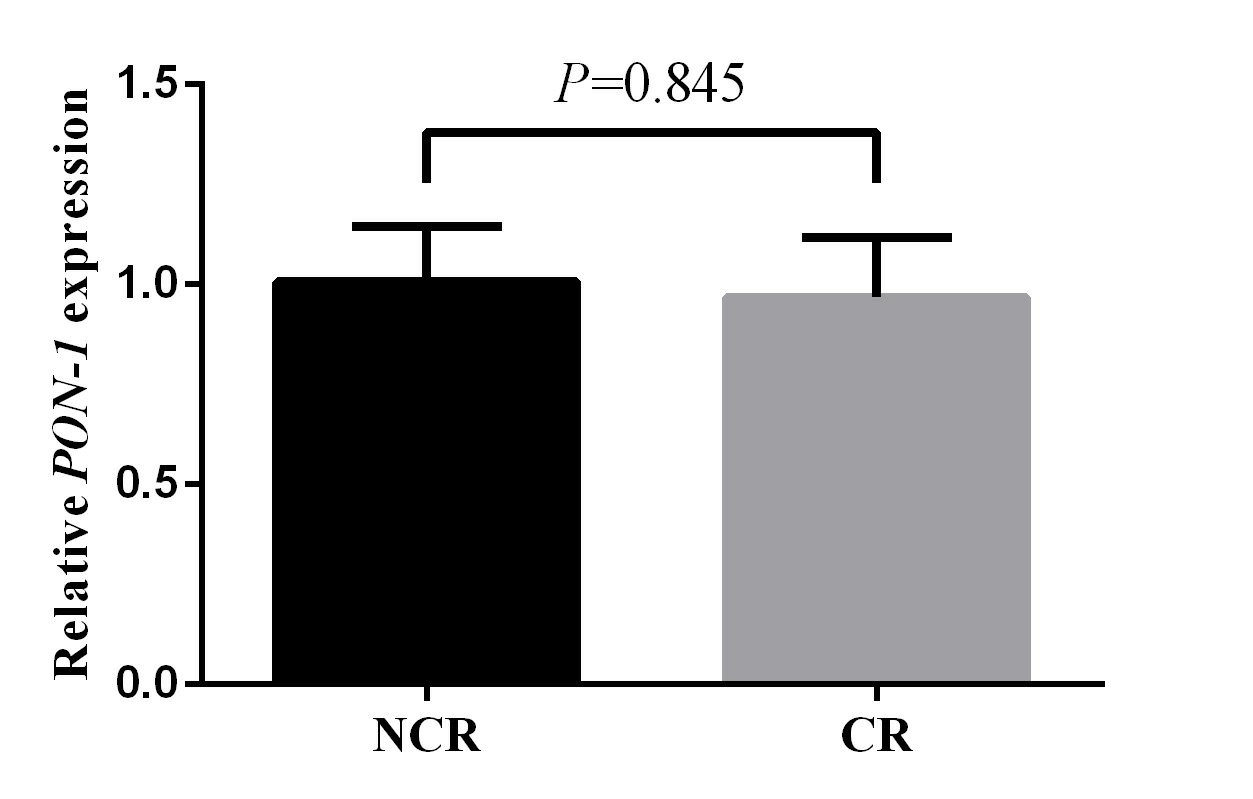


Figure S2 A comparison of PON1 mRNA expression between cases and controls
